# COVID-19, Food Insecurity and Malnutrition: A Multiple Burden for Brazil

**DOI:** 10.3389/fnut.2021.751715

**Published:** 2021-12-15

**Authors:** Rita de Cássia Ribeiro-Silva, Marcos Pereira, Érika Aragão, Jane Mary de Medeiros Guimarães, Andrêa J. F. Ferreira, Aline dos Santos Rocha, Natanael de Jesus Silva, Camila Silveira Silva Teixeira, Ila Rocha Falcão, Enny Santos Paixao, Mauricio Lima Barreto

**Affiliations:** ^1^Departamento Ciência da Nutrição, Escola de Nutrição, Universidade Federal da Bahia – Salvador, Bahia, Brazil; ^2^Centro de Integração de Dados e Conhecimentos para Saúde (CIDACS), Fundação Oswaldo Cruz – FIOCRUZ, Salvador, Bahia, Brazil; ^3^Instituto de Saúde Coletiva (ISC), Universidade Federal da Bahia – Salvador, Bahia, Brazil; ^4^Universidade Federal do Sul da Bahia (UFSB) – Itabuna, Bahia, Brazil; ^5^Faculty of Epidemiology and Population Health, London School of Hygiene and Tropical Medicine, London, United Kingdom

**Keywords:** COVID-19, social inequality, social distancing, overweight, obesity, food consumption, social isolation (SI)

## Introduction

The spread of COVID-19 has triggered a severe global economic crisis and led to political, economic and social consequences, according to the World Bank (1). In addition to the ever-increasing numbers of COVID-19 cases and deaths, as well as considerable stress to health systems, governments also face economic destabilization due to compromised income generation and limited access to resources (e.g., employment, food, housing, and healthcare) for substantial segments of populations worldwide (2).

In low- and middle-income countries, the COVID-19 pandemic is likely to precipitate some degree of food insecurity in socioeconomically vulnerable populations facing budget constraints (3). Individuals living in households struggling with food insecurity generally do not have adequate food access in terms quantity and quality (4). A nutritionally deficient diet results in a series of vulnerabilities, often leading to nutrition-related morbidities with a spectrum of manifestations (5). A diet low in complex carbohydrates and rich in simple sugars and fats has been associated with an increased risk of developing or exacerbating diet-related non-communicable diseases (NCD), including obesity, cardiovascular disease, diabetes and certain types of cancer, as well as premature mortality (4). An increasing burden of malnutrition, a predictable consequence, was already estimated in the global scenario even before the pandemic (6, 7) and children, pregnant and lactating women could be more affected (8).

Estimates published in the United Nations Food and Agriculture Organization (FAO) 2020 report (7) indicate the world is not on track to meet global nutrition goals, such as reducing the incidence of low birth weight and stunting among children aged 5 years or younger. In addition, the prevalence of overweight and obesity continues to increase worldwide, especially among schoolchildren (7). The potential impacts of COVID-19 could further aggravate this scenario, especially in core and periphery countries that have inadequately responded to the burden of the pandemic (9). Economic fragility in these countries has led to more severe loss of income, resulting from increased informality and instability in employment, and the implementation of neoliberal agendas has pushed thousands into extreme poverty. This condition jeopardizes the survival of those who are unable to secure livelihood, consequently violating the human right of access to a sufficient quantity of nutritious and safe food (10, 11).

In Brazil, the COVID-19 pandemic has had a disastrous impact, as evidence by more than 600,000 deaths, increased social inequality and hunger (12). As a result, the consequences of the pandemic deserve ample discussion to support public interventions aimed at fighting the pandemic and its consequences. This opinion paper aims to analyze the risks and consequences associated with an increasing prevalence of malnutrition in Brazil due to the COVID-19 pandemic. Here, we also systematize strategies to protect vulnerable populations not only from the effects of malnutrition, but also from profound inequities in food and financial security in the coming years.

## Social Inequality and Food Insecurity in Brazil

In Brazil, the association between social inequality and unhealthy dietary patterns places vulnerable populations at serious risk of food insecurity (availability, accessibility, affordability, and utilization of foods), undernutrition and obesity (13, 14). Brazil remains one of the countries with the highest rates of income inequality in the world, despite significant progress made over the last two decades in reducing poverty and improving socioeconomic and health conditions (14). However, this trend reversed in 2015, as evidenced by recent reports of increases in poverty and inequality. In 2019, 1% of the country's highest-income population earned 33.7 times more than those comprising the 50% lower income bracket (15). This data exposes one of the most structural aspects of inequality in Brazil—the income gap between rich and poor.

The health crisis provoked by COVID-19, which has resulted in pronounced economic and social impacts worldwide, hit Brazil at a time of labor market disruption, with rising unemployment and precariousness in the social protection network (16). The adoption of strategies to reduce and control infection rates has had a particularly huge impact on economic markets, leading to significant disruptions in food availability and accessibility—two pillars of a stable food security system (17). As a result, household consumption expenditures plummeted, especially among the poorest, according to the Brazilian Institute of Geography and Statistics (IBGE) (18). Thus, food insecurity resulting from income restrictions, which make access to nutritionally adequate and healthy foods more difficult, has advanced and continues to intensify in the country. Before the COVID-19 pandemic, 36.7% of the 68.9 million households in Brazil experienced some degree of food insecurity, impacting 84.9 million people (19). By 2020, this figure increased dramatically across the country, particularly among those at higher risk of malnutrition. However, it is known that the individuals most vulnerable to the food and nutrition crisis caused by COVID-19 are those who were exposed to food deprivation even before the pandemic (15). The number of people living in extreme poverty in Brazil increased by 170,000 in 2019 to reach 13.8 million, equivalent to 6.7% of the country's population (18). As of June 2021, almost 15 million Brazilians were unemployed, and almost 3.5 million of those individuals had been jobless for over 2 years (20).

The main reasons underlying this trend are 3 fold: The discontinuity and poor execution of social protection programs, such as the Brazilian cash transfer program (Bolsa Família), which has suffered budgetary cuts every year since 2014; the devaluation of the country's minimum monthly wage policy, which is key to preserving the low-income population's purchasing power; the disintegration of Brazilian labor law reform (CLT), which has generated sharp increases in informal employment and job insecurity (21). Thus, fiscal austerity and the federal government's liberal reform agenda, which imposed severe budget restrictions on social programs as well as the national unified health system (SUS), has provoked a desolate scenario that severely impacted the income of the country's most vulnerable families, and also exacerbated food insecurity and hunger across millions of Brazilian households nationwide (22). This dismantlement, more so than the COVID-19 pandemic, underlies Brazil's return to the UN Hunger Map (23). Unfortunately, hunger has become an ever-increasing daily burden for many families residing on the outskirts of large cities, the homeless and more families living in isolated rural areas, including indigenous and *quilombola* populations (15, 24, 25).

Together, this information reveals that the dire situation regarding food and nutrition insecurity in the country cannot be completely attributed to the pandemic. Indeed, the previously existing scenario of unresolved social inequalities, in addition to the advancement of neoliberal policies and the dissolution of important social development and food and nutrition security programs, has been aggravated by the current crisis, which has become worse due to the impacts of the COVID-19 pandemic.

## Food Insecurity and Malnutrition in the Context of a Pandemic

Pandemic-related crises and social distancing policies have contributed to disproportionally increase poverty and food insecurity in the Brazilian population (23). The most damaging impact brought by food insecurity on individual health is malnutrition. Although commonly associated with undernutrition (i.e., wasting, stunting, underweight), malnutrition is also recognized as a consequence of overnutrition and characterized by diet-related NCDs (e.g., obesity, cardiovascular disease, diabetes, certain types of cancer). Both nutritional conditions may be affected by food prices. During the pandemic, heathier food and diets have become more expensive and increasingly limited (26). Fewer resources forces the population to turn to cheaper foods, which are mostly ultra-processed, rich in sugar, sodium and fat while lacking nutrients (4, 27). The problem is that these items—which tend to be more caloric and less nutritious than fresh foods—may, over the medium term, place consumers at increasing risk for the double burden of malnutrition (15, 27, 28).

It is known that limited access to food and rates of poverty are intricately linked, i.e., in most cases, food insecurity and hunger are not necessarily related to the unavailability of food, but rather to insufficient income needed to acquire it (15). Brazilian currency devaluation, as reflected by the increased price of the US dollar, combined with the country's lack of food supply planning (food stocks are not regulated) has raised food prices. Another consideration is that by lacking incentives, differentiated lines of credit and mechanisms to support the marketing of family farm products—responsible for 70% of the fresh food supply—the flow/marketing of fresh and healthy food has become compromised, thus forcing higher prices. This set of limitations has prevented regular and permanent access to sufficient quantities of quality food, essential to promoting healthy weight, especially among the poorest populations (29, 30).

This scenario raises relevant concerns for both the Brazilian public health system and society as a whole not only during, but also after the COVID-19 pandemic. The adoption of healthy and natural dietary habits is known to aid in the prevention of COVID-19. In addition to providing essential vitamins, macro- and micronutrients and bioactive compounds necessary to maintain the integrity of the immune barrier, healthy eating habits can also ensure the maintenance of adequate weight; both malnutrition and obesity have been associated with worse outcomes in COVID-19 patients, linked to higher rates of hospitalization, longer hospital stays and greater risk of mortality (31), (32, 33). Thus, proactive responses to food and nutrition insecurity, such as the implementation of strategies designed to guarantee proper access to food among the most vulnerable populations, must be considered to maintain resilience and mitigate the pandemic's impacts on health and nutrition. It is urgent for governmental authorities to consider resuming the emergency cash transfers prompted by COVID-19, as well as to contemplate previous administrations' past success in fighting hunger.

## Strategy to Promote Healthy Diets and Curb Malnutrition in the Time of a Pandemic

The need to adopt strategies to slow the spread of SARS-CoV-2 infection is urgent, as are efforts to protect obese individuals and implement actions to mitigate malnutrition over the short-, medium- and long-term. It is also necessary to address some other determinants of an unhealthy diet, such as social inequality, which are likely to compound challenges associated with controlling the COVID-19 epidemic in Brazil (14). Thus, there can be no doubt that protecting income is extremely necessary to guarantee adequate access to the resources needed to achieve and maintain a healthy weight. In addition, the spheres of power must also create structured and reliable support systems to guarantee the availability of and access to affordable essential food items for all households, which is crucial to ensuring that families can put safe and healthy food on their tables. In places where the marketplace has reopened, strategies that mix the use of vouchers and assistance provided by social welfare programs can facilitate more immediate access to food.

## Conclusions

The challenge Brazil faces now is to use a variety of mechanisms to leverage different food supply strategies to support access to food, a social right included in Article 6 of the Federal Constitution (in February 2010) (34). However, the longstanding international human right to adequate food is a distant reality for many worldwide, and the COVID-19 pandemic has accentuated previously existing challenges. In this context, the strengthening of the Food Acquisition Program (PAA), created in 2003 by the Ministry of Citizenship to promote access to food and incentivize family farming (notably the modalities of direct procurement and procurement with simultaneous donation) and the continued operationalization of the National School Meals Program (PNAE), an initiative of the Ministry of Education that provides all grades of children in public schools with food and nutritional education (adjusted to conform to COVID-19 restrictions) are some measures that should be immediately implemented (35, 36). Structural programs, such as the Bolsa Família and Continuous Benefit Programs (BPC: the Non-contributory Regular Pension), the latter of which provides financial support to impoverished elderly persons and those with disabilities, should be considered emergency response strategies to the present crisis. These initiatives have the ability to rapidly provide income to families facing vulnerable socioeconomic situations. Moreover, the continuity, expansion and systematization of food distribution (in low-cost public restaurants, food banks, community soup kitchens and family farming distribution units), adapted to observe sanitary precautions to reduce SARS-CoV-2 dissemination risk, are of paramount importance as an urgent strategy to alleviate hunger in several vulnerable groups across Brazil. We further recommend the implementation of food and nutrition education initiatives (i.e., educational programs on television, over the internet or radio) to guide and encourage the adoption/maintenance of healthy eating habits for all family members. Logically, the monitoring of nutritional status should also be considered.

## Author Contributions

RR-S conceived and wrote the manuscript. MP, AF, AR, NS, CT, and IF contributed to the manuscript elaboration. ÉA, JG, EP, and MB critically revised the text. All authors have read and approved the final version of the manuscript.

## Conflict of Interest

The authors declare that the research was conducted in the absence of any commercial or financial relationships that could be construed as a potential conflict of interest.

## Publisher's Note

All claims expressed in this article are solely those of the authors and do not necessarily represent those of their affiliated organizations, or those of the publisher, the editors and the reviewers. Any product that may be evaluated in this article, or claim that may be made by its manufacturer, is not guaranteed or endorsed by the publisher.

## References

[1] COVID-19 to Plunge Global Economy into Worst Recession since World War II. Available online at: https://www.worldbank.org/en/news/press-release/2020/06/08/covid-19-to-plunge-global-economy-into-worst-recession-since-world-war-ii (accessed November 19, 2020).

[2] Nicola M Alsafi Z Sohrabi C Kerwan A Al-Jabir A Iosifidis C. The socio-economic implications of the coronavirus pandemic (COVID-19): a review. Int J Surg. (2020) 78:185–93. 10.1016/j.ijsu.2020.04.018 32305533 PMC7162753

[3] Kinsey EW Kinsey D Rundle AG. COVID-19 and food insecurity: an uneven patchwork of responses. J Urban Health. (2020) 97:332–5. 10.1007/s11524-020-00455-5 32504251 PMC7274516

[4] Huizar MI Arena R Laddu DR. The global food syndemic: the impact of food insecurity, malnutrition and obesity on the healthspan amid the COVID-19 pandemic. Progr Cardiovasc Dis. (2021) 64:105–7. 10.1016/j.pcad.2020.07.002 32653438 PMC7347484

[5] Dinour LM Bergen D Yeh MC. The food insecurity-obesity paradox: a review of the literature and the role food stamps may play. J Am Dietetic Assoc. (2007) 107:1952–61. 10.1016/j.jada.2007.08.006 17964316

[6] Swinburn BA Kraak VI Allender S Atkins VJ Baker PI Bogard JR. The global syndemic of obesity, undernutrition, and climate change: the lancet commission report. Lancet. (2019) 393:791–846. 10.1016/S0140-6736(18)32822-8 30700377

[7] The State of Food Security and Nutrition in the World 2020. FAO, IFAD, UNICEF, WFP and WHO (2020). Available online at: http://www.fao.org/documents/card/en/c/ca9692en (accessed November 19, 2020).

[8] Roberton T Carter ED Chou VB Stegmuller AR Jackson BD Tam Y. Early estimates of the indirect effects of the COVID-19 pandemic on maternal and child mortality in low-income and middle-income countries: a modelling study. Lancet Glob Health. (2020) 8:e901–8. 10.1016/S2214-109X(20)30229-1 32405459 PMC7217645

[9] Morales ME Berkowitz SA. The Relationship between food insecurity, dietary patterns, and obesity. Curr Nutr Rep. (2016) 5:54–60. 10.1007/s13668-016-0153-y 29955440 PMC6019322

[10] Atar S Atar I. An invited commentary on “The socio-economic implications of the coronavirus and COVID-19 pandemic: a review”. Int J Surg. (2020) 78:122. 10.1016/j.ijsu.2020.04.054 32360501 PMC7189197

[11] Naja F Hamadeh R. Nutrition amid the COVID-19 pandemic: a multi-level framework for action. Eur J Clin Nutr. (2020) 74:1117–21. 10.1038/s41430-020-0634-3 32313188 PMC7167535

[12] Ministério da Saúde (Brasil),. Secretaria de Atenção à Saúde. Coronavírus Brasil. (2021). Available online at: https://covid.saude.gov.br/ (accessed October 25, 2021).

[13] Ribeiro-Silva RC Pereira M Campello T Aragao E Guimaraes JMM Ferreira AJ. Covid-19 pandemic implications for food and nutrition security in Brazil. Cien Saude Colet. (2020) 25:3421–30. 10.1590/1413-81232020259.22152020 32876253

[14] Alpino TMA Santos CRB Barros DC Freitas CM. COVID-19 and food and nutritional (in)security: action by the Brazilian Federal Government during the pandemic, with budget cuts and institutional dismantlement. Cad Saude Publica. (2020) 36:e00161320. 10.1590/0102-311x00161320 32901703

[15] Instituto Brasileiro de Geografia e Estatística. Available online at: https://agenciadenoticias.ibge.gov.br/en/agencia-press-room/2185-news-agency/releases-en/27601-continuous-pnad-2019-earnings-form-the-1-higher-income-classes-correspond-to-33–7-times-the-earnings-of-half-of-lower-income-classes (accessed November 19, 2020).

[16] Hone T Mirelman AJ Rasella D Paes-Sousa R Barreto ML Rocha R. Effect of economic recession and impact of health and social protection expenditures on adult mortality: a longitudinal analysis of 5565 Brazilian municipalities. Lancet Glob Health. (2019) 7:e1575–83. 10.1016/S2214-109X(19)30409-7 31607469

[17] Pereira M Oliveira AM. Poverty and food insecurity may increase as the threat of COVID-19 spreads. Public Health Nutr. (2020) 23:3236–40. 10.1017/S1368980020003493 32895072 PMC7520649

[18] Instituto, Brasileiro de Geografia e Estatística,. Pesquisa Nacional por Amostra de Domicílios Contínua - PNAD Contínua. IBGE. Available online at: https://www.ibge.gov.br/estatisticas/sociais/populacao/9171-pesquisa-nacional-por-amostra-de-domicilios-continua-mensal.html?=&t=o-que-e (accessed November 19, 2020).

[19] Instituto, Brasileiro de Geografia e Estatística,. 10.3 Million People Live in Households With Severe Food Insecurity. Available online at: https://agenciadenoticias.ibge.gov.br/agencia-noticias/2012-agencia-de-noticias/noticias/28903–10-3-milhoes-de-pessoas-moram-em-domicilios-com-inseguranca-alimentar-grave (accessed November 19, 2020).

[20] Instituto Brasileiro de Geografia e Estatística,. Pesquisa Nacional por Amostra de Domicílios Contínua Primeiro Trimestre de 2021. Rio de Janeiro: IBGE (2021). Available online at https://biblioteca.ibge.gov.br/visualizacao/periodicos/2421/pnact_2021_1tri.pdf (accessed June 18, 2021).

[21] de Souza L de Barros RD Barreto ML Katikireddi SV Hone TV Paes de Sousa R. The potential impact of austerity on attainment of the sustainable development goals in Brazil. BMJ Glob Health. (2019) 4:e001661. 10.1136/bmjgh-2019-001661 31565412 PMC6747892

[22] Rasella D Basu S Hone T Paes-Sousa R Ocke-Reis CO Millett C. Child morbidity and mortality associated with alternative policy responses to the economic crisis in Brazil: a nationwide microsimulation study. PLoS Med. (2018) 15:e1002570. 10.1371/journal.pmed.1002570 29787574 PMC5963760

[23] Carvalho CA Viola P Sperandio N. How is Brazil facing the crisis of Food and Nutrition Security during the COVID-19 pandemic? Public Health Nutr. (2021) 24:561–4. 10.1017/S1368980020003973 33040767 PMC7642956

[24] Santos RV Pontes AL Coimbra CEA Jr. A “total social fact”: COVID-19 and indigenous peoples in Brazil. Cad Saude Publica. (2020) 36:e00268220. 10.1590/0102-311X00268220 33027432

[25] Paula HC Daher DV Koopmans FF Faria MGA Lemos PFS Moniz MA. No place to shelter: ethnography of the homeless population in the COVID-19 pandemic. Rev Bras Enferm. (2020) 73(Suppl. 2):e20200489. 10.1590/0034-7167-2020-0489 33206817

[26] Adams EL Caccavale LJ Smith D Bean MK. Food insecurity, the home food environment, and parent feeding practices in the era of COVID-19. Obesity. (2020) 28:2056–63. 10.1002/oby.22996 32762129 PMC7436743

[27] Smaira FI Mazzolani BC Esteves GP Andre HCS Amarante MC Castanho DF. Poor eating habits and selected determinants of food choice were associated with ultraprocessed food consumption in brazilian women during the COVID-19 pandemic. Front Nutr. (2021) 8:672372. 10.3389/fnut.2021.672372 34055859 PMC8155283

[28] Allard L Ouedraogo E Molleville J Bihan H Giroux-Leprieur B Sutton A. Malnutrition: percentage and association with prognosis in patients hospitalized for coronavirus disease 2019. Nutrients. (2020) 12:3679. 10.3390/nu12123679 33260603 PMC7761464

[29] Hohler J Lansink AO. Measuring the impact of COVID-19 on stock prices and profits in the food supply chain. Agribusiness. (2020) 37:171–86. 10.1002/agr.21678 33362339 PMC7753738

[30] Silva Filho OJD Gomes Junior NN. The future at the kitchen table: COVID-19 and the food supply. Cad Saude Publica. (2020) 36:e00095220. 10.1590/0102-311x00095220 32490915

[31] Kurtz A Grant K Marano R Arrieta A Grant K Jr Feaster W. Long-term effects of malnutrition on severity of COVID-19. Sci Rep. (2021) 11:14974. 10.1038/s41598-021-94138-z 34294743 PMC8298504

[32] Pironi L Sasdelli AS Ravaioli F Baracco B Battaiola C Bocedi G. Malnutrition and nutritional therapy in patients with SARS-CoV-2 disease. Clin Nutr. (2021) 40:1330–7. 10.1016/j.clnu.2020.08.021 32900518 PMC7450234

[33] Leddy AM Weiser SD Palar K Seligman H. A conceptual model for understanding the rapid COVID-19-related increase in food insecurity and its impact on health and healthcare. Am J Clin Nutr. (2020) 112:1162–9. 10.1093/ajcn/nqaa226 32766740 PMC7454255

[34] Brasil. Lei n° 11.346, de 15 de Setembro de 2006. Cria o Sistema Nacional de Segurança Alimentar e Nutricional – SISAN com Vistas em Assegurar o Direito Humano à Alimentação Adequada e dá Outras providências. Diário Oficial da União, Brasília (DF) (2006).

[35] Sambuichi RHR Almeida AFCS Perin G Spínola PAC Pella AFC. The Food Acquisition Program (PAA) as a strategy to face the challenges of COVID-19. Rev Adm Pública. (2020) 54:1079–96. 10.1590/0034-761220200258x

[36] Brasil. Lei n° 13.987, de 7 de Abril de 2020. Altera a Lei n° 11.947, de 16 de Junho de 2009, Para Autorizar, em Caráter Excepcional, Durante o Período de Suspensão das Aulas em Razão de Situação de Emergência ou Calamidade Pública, a Distribuição de Gêneros Alimentícios Adquiridos com Recursos do Programa Nacional de Alimentação Escolar (PNAE) aos pais ou Responsáveis dos Estudantes das Escolas Públicas de educação Básica. Diário Oficial da União, Brasília (DF) (2020).

